# Preparation of DNA functional phosphorescent quantum dots and application in melamine detection in milk†

**DOI:** 10.1039/c9ra03919h

**Published:** 2019-07-05

**Authors:** Yanming Miao, Ruirui Wang, Xiaojie Sun, Guiqin Yan

**Affiliations:** Shanxi Normal University Linfen 041004 PR China mym8207@126.com +86-357-2051243 +86-357-2051249

## Abstract

Bio-functionalization of quantum dots (QDs) is of important value in practical applications. With single-stranded DNA (ssDNA) rich in thymine T and thioguanine G taken as the template, a new-type nanocomposite material (ssDNA-PQDs) synthesized from low-toxicity T-ssDNA functionalized Mn–ZnS and room-temperature phosphorescent (RTP) QDs (PQDs) was prepared in this paper by optimizing synthesis conditions, and these ssDNA-PQDs could emit orange RTP signals at 590 nm. As these ssDNA-PQDs are rich in T sequences and T sequences can bond with melamine through the hydrogen-bond interaction, ssDNA-PQDs experience aggregation, thus causing phosphorescent exciton energy transfer (PEET) between ssDNA-PQDs of different particle sizes and their RTP quenching. Based on this principle, an RTP detection method for melamine was established. The linear range and detection limit of the detection method are 0.005–6 mM and 0.0016 mM respectively. As this method is based on the RTP nature of ssDNA-PQDs, it can avoid disturbance from background fluorescence and scattered light of the biological fluid, and it is very suitable for melamine detection in the biological fluid milk.

## Abstract 2

Preparation of phosphorescent quantum dots taking single-stranded DNA as a template and their application to melamine detection in milk.

## Graphical abstract

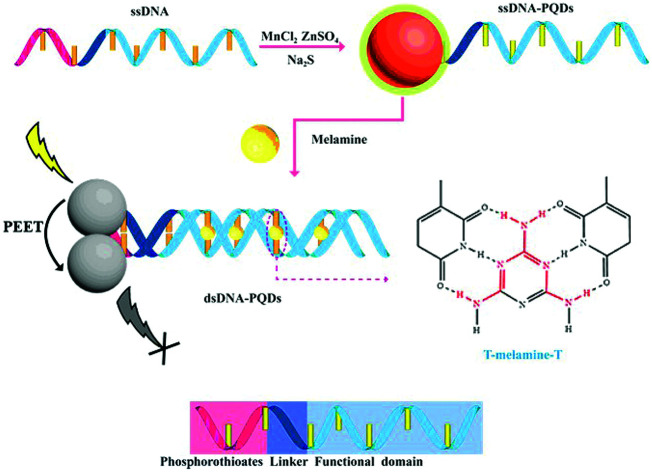

## 1. Introduction

As bio-functionalized (protein, DNA, *etc.*) QDs^1–3^ feature favorable biocompatibility, low toxicity and degradability, *etc.*, they are of important application value in fields like biomolecular sensing,^4^ environmental pollutant detection^5^ and biomedicine,^6^ so they have been extensively applied in practice.^7,8^ Especially DNA molecules^9–11^ have many advantages, including favorable thermal stability and an electronegative pentose-phosphate backbone which can easily bond with metal ions.^12–14^ Moreover, they will not harm the environment after degradation and can artificially synthesize DNAs of specific sequences and form many aptamers of different sequences on QDs surfaces, which can contribute to preparation of multiple kinds of discriminatory analysis sensors of good selectivity. Therefore, DNA functionalized QDs have an extensive application prospect.^1,15^ Even though bio-functionalized QDs have achieved a certain progress in the past years, the interface connection between controllable hydrophilic biomolecules and QDs of inorganic surfaces is still an important research direction, especially as there is little research on PQDs synthesis using DNA as the template.

As PQDs^16–18^ not only have advantages such as long fluorescence lifetime, high selectivity, broad linearity range, high sensitivity and good repeatability but also can avoid disturbance from background fluorescence and scattered light of the biological fluid,^19,20^ especially Mn–ZnS QDs, which have superior optical properties, have been widely applied in the biomolecular detection process.^21,22^ If DNA-PQDs are prepared using DNA as the template, this will greatly widen the application range of existing Mn–ZnS PQDs in biology.

As a low-toxicity industrial raw material,^23^ melamine (M) is a new-type pollutant appearing in milk, infant formula milk powder and pet food.^24^ In order to increase “fake” protein contents in food, melamine is artificially and illegally used as non-protein nitrogen additive. Melamine contained in milk will be absorbed by human body and can cause kidney diseases and even death of infants and children,^25–28^ so melamine detection in milk will be of important application value to milk safety evaluation.

**Fig. 1:**
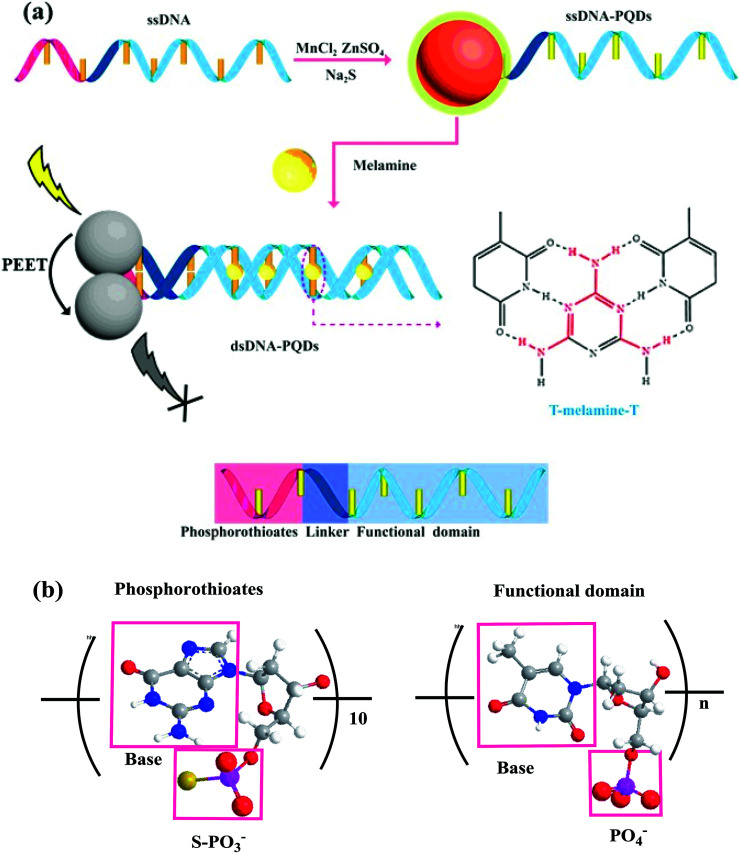
(a) Schematic diagram of ssDNA-PQDs preparation and their interaction with melamine; (b) structural diagram of thiophosphate and functional domain.

ssDNA rich in thymine T and thioguanine G taken as the template and Mn^2+^, Zn^2+^ and S^2−^ taken as raw materials, ssDNA-PQDs were prepared in this paper. As this ssDNA template included three structural domains namely thiophosphoric acid ester, linking group and functional domain, and its one end contained PS guanine G nucleotide related to QDs synthesis while the other end was T basic group ssDNA sequence which can identify melamine, specific identification ability of T basic group for melamine was utilized to realize RTP detection of melamine. The construction process is shown in Fig. 1. The compound was generated by Zn^2+^ and Mn^2+^ through the biomineralization effect together with PS guanine G nucleotide. After S^2−^ was added, PQDs of T basic group ssDNA sequence functionalization were further generated. The melamine detection principle in milk using these ssDNA-PQDs is that T basic group of ssDNA-PQDs can go through hydrogen-bond reaction with melamine to form T–M–T conjugate, which causes mutual aggregation of ssDNA-PQDs, causing PEET between ssDNA-PQDs of different particle sizes and their RTP quenching.

On this basis a selectable RTP sensor identifying melamine was constructed (Fig. 1).

## 2. Experimental

### 2.1 Materials and reagents

ssDNA used for preparation was purchased from Sangon Biotech (Shanghai) Co., Ltd. MnCl_2_·4H_2_O, ZnSO_4_·7H_2_O and Na_2_S·9H_2_O were purchased from Tianjin Kermel Chemical Reagents Co., Ltd. Melamine was purchased from Beijing Bailing Way Technology Co., Ltd. High-purity water was prepared using WaterPro Water Purification System (US Labconco Corporation).

### 2.2 Main instruments

ssDNA-PQDs were tested using JEM-2100TEM device (transmission electron microscope) (Japan Electron Optics Lab Co., Ltd, JEOL); phosphorescent spectrum analysis was carried out using Cary Eclipse fluorescence spectrophotometer; the absolute quantum yield of ssDNA-PQDs was tested using Edinburgh FLS1000 fluorescence spectrometer; UV spectra were determined using Shimadzu UV-29100 UV spectrophotometer; solution pH was determined using pHS3C pH meter; surface elemental composition of ssDNA-PQDs was characterized on Thermo escalab 250Xi X-ray photoelectron spectrometer.

### 2.3 PQDs synthesis taking single-stranded DNA as the template

8 μL 0.074 mM MnCl_2_, 39.6 μL 7.3 mM ZnSO_4_ and 30 μL 0.48 μM ssDNA were added into the Tris-HCl buffer solution with pH = 7.0. The solution was heated in water bath to 50 °C and then sealed for oxidative reaction for 5 min after shaken up; subsequently, 36 μL 6.7 mM Na_2_S was added into the abovementioned solution and its constant volume was turned to 270 μL; ssDNA-PQDs were obtained after 30 min reaction of the solution under sealing conditions.

### 2.4 Calculation method of the absolute quantum yield of ssDNA-PQDs

The computation of absolute quantum yield avoided the use of standard substances and returned relatively small measurement errors. Firstly, a stable monochromatic LED at appropriate wavelength (emission wavelength at 400–450 nm) was selected as the excitation source. Then the samples of ssDNA-PQDs were progressively diluted to a concentration gradient. The absolute quantum yields were measured after the diluted solutions were balanced and stable. The excitation source was opened, and the blank solution and the ssDNA-PQDs solutions placed in little bottles were separately put into optical integral balls. At this moment, the excitation photons and the emission fluorescence photons after absorption underwent a series of reflection and absorption in the optical integral balls and were finally transferred *via* optical fibers into the fluorescence spectrometer. The counts of excitation photons and the emission fluorescence photons after the absorption were recorded. The absolute quantum yield was calculated as: *ψ* = quantity of consumed reactants (or products)/count of absorbed photons.

### 2.5 Determination method of melamine

In order to study the influence of melamine on RTP of ssDNA-PQDs, melamine was dissolved in water to form 10 mM water solution. 500 μL buffer solution (PBS, pH 8.0, 0.2 M), 50 μL ssDNA-PQDs solution and melamine solutions of different concentrations were added into the colorimetric tube in succession, the mixture was fixed to volume 5 mL and then blended, and 20 min later, RTP was detected (*λ*_ex_ = 297 nm), and the experiment was repeated for three times.

### 2.6 Interference experiment

In order to study the interference of ssDNA-PQDs in detecting melamine, 500 μL buffer solution (PBS, pH 8.0, 0.2 M), 50 μL ssDNA-PQDs, 20 μM melamine and interfering substance (different concentrations of K^+^, Na^+^, Mg^2+^, Ca^2+^, Zn^2+^, Hg^2+^, Ag^+^, Cu^2+^, Pb^2+^, Co^2+^, glucose (Glc), l-alanine (l-Ala), l-lysine (l-Lys), l-tyrosine (l-Tyr) and l-glutamic acid (l-Glu)) were added into the corresponding colorimetric tube successively, the mixture was fixed to volume 5 mL using secondary water and then shaken up, RTP was detected 20 min later (*λ*_ex_ = 297 nm), and the experiment was repeated for three times.

### 2.7 Melamine detection in milk

PBS (pH 8.0, 0.2 M, 500 μL), ssDNA-PQDs solution (50 μL), milk sample and melamine of different concentrations (10 μM, 50 μM and 100 μM) were added into the 10 mL colorimetric tube in succession, the mixture was fixed to volume 5 mL and shake up, RTP was detected 20 min later (*λ*_ex_ = 297 nm), and the experiment was repeated for three times. Except for being diluted by 50 times, the milk sample didn't go through any other pretreatment.

## 3. Results and discussion

### 3.1 Factors influencing ssDNA-PQDs stability

**Fig. 2:**
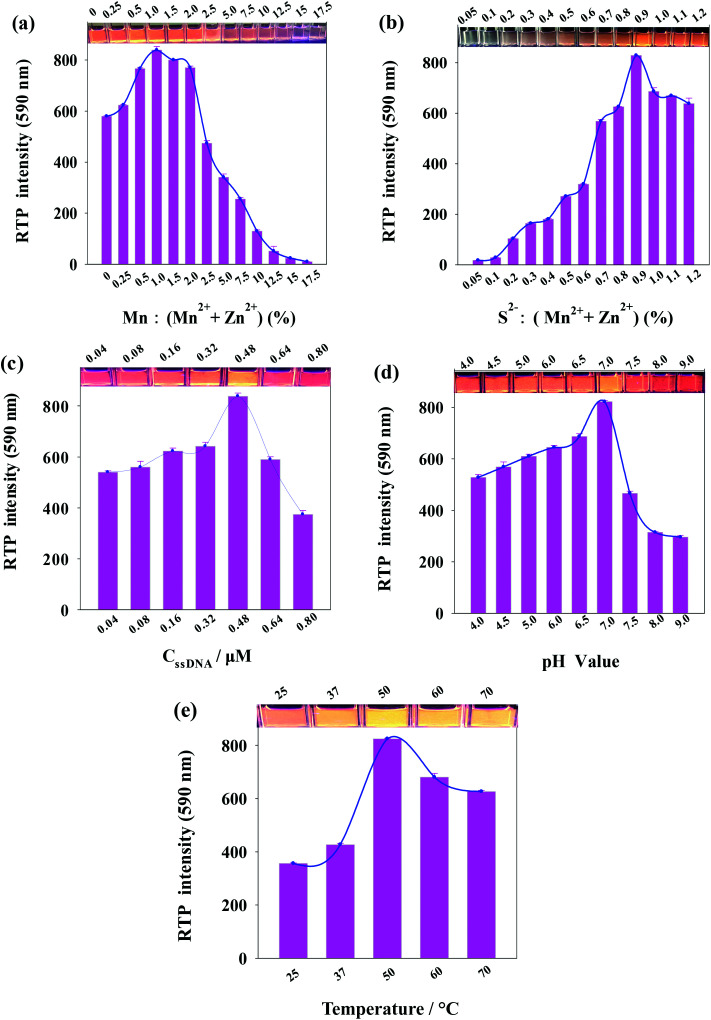
(a) The influence of Mn^2+^ : (Mn^2+^ + Zn^2+^) on ssDNA-PQDs RTP intensity; (b) the influence of S^2−^ : (Mn^2+^ + Zn^2+^) on ssDNA-PQDs RTP intensity; (c) the influence of ssDNA concentration on ssDNA-PQDs RTP intensity; (d) the influence of pH value on ssDNA-PQDs RTP intensity; (e) the influence of temperature on ssDNA-PQDs RTP intensity (*λ*_ex_ = 297 nm, *λ*_em_ = 590 nm).

As phosphorescent property of ssDNA-PQDs is closely related to the molar concentration ratio of reactants, ssDNA concentration, pH value and reaction temperature, these factors were optimized respectively during the ssDNA-PQDs preparation process. As shown in Fig. 2(a), the Mn^2+^ : (Mn^2+^ + Zn^2+^) molar ratio was optimized. The RTP intensity of ssDNA-PQDs was gradually enhanced when Mn^2+^ : (Mn^2+^ + Zn^2+^) was within 0–1.0%, maximized at molar ratio of 1.0, and it gradually dropped when Mn^2+^ : (Mn^2+^ + Zn^2+^) was within the range of 1.0–17.5%. When Mn^2+^ was excessive, RTP was decreased due to the absorption of excess Mn^2+^ on the surface of ssDNA-PQDs to capture electrons, thereby resulting in RTP quenching.^29^

Fig. 2(b) shows the variation in the RTP intensity of ssDNA-PQDs along with the S^2−^ : (Mn^2+^ + Zn^2+^) molar ratio. The RTP intensity of ssDNA-PQDs was gradually enhanced when S^2−^ : (Mn^2+^ + Zn^2+^) was within the range of 0.05–0.9%, maximized at molar ratio of 0.9, and then gradually dropped with the further increase in molar ratio. When S^2−^ was excessive, RTP was decreased due to the excess S^2−^ that captured holes on the surface of ssDNA-PQDs, thereby resulting in RTP quenching.^30^

In Fig. 2(c), the ssDNA concentration was optimized. The RTP intensity of ssDNA-PQDs was gradually enhanced when ssDNA concentration was within the range of 0.04–0.48 μM, maximized at ssDNA concentration of 0.48 μM, and then gradually dropped with further increase in ssDNA-PQDs concentration. Since the oxidation potential of guanine (G base) (1.29 V *versus* the standard hydrogen electrode) is lower than that of adenine (1.42 V) or other pyrimidines, guanine is more prone to electron transfer or namely has stronger surface negativity.^31^ The ssDNA used in our study was rich in G bases, and thus when ssDNA was excessive, the G bases of ssDNA will oxidize ssDNA-PQDs, which led to lower RTP and thereby the RTP quenching of ssDNA-PQDs.

Fig. 2(d) shows the variation in the RTP intensity of ssDNA-PQDs along with the pH in the synthesis system. The RTP intensity of ssDNA-PQDs was gradually enhanced when pH value was within the range of 4.0–7.0, maximized when pH value was 7.0, and then gradually dropped when the pH further increase. The possible reason was that the strong Zn^2+^ and ssDNA interaction at pH 7.0 can well modify the surface defects of nanoparticles and convert the nonradiative electron–hole recombination into radiative recombination, which led to the strong RTP.

In Fig. 2(e), the reaction temperature was optimized. The RTP intensity of ssDNA-PQDs was gradually enhanced when the reaction temperature was within the range of 25–50 °C, maximized when the reaction temperature was 50 °C, and it gradually dropped within the range of 50–75 °C.

These results suggested that the optimal conditions for ssDNA-PQDs synthesis are as follows: Mn^2+^ : (Mn^2+^ + Zn^2+^) molar ratio = 1.0%, S^2−^ : (Mn^2+^ + Zn^2+^) molar ratio = 0.9%, ssDNA concentration = 0.48 μM, pH = 7.0, and reaction temperature = 50 °C.

### 3.2 ssDNA-PQDs characterization

**Fig. 3:**
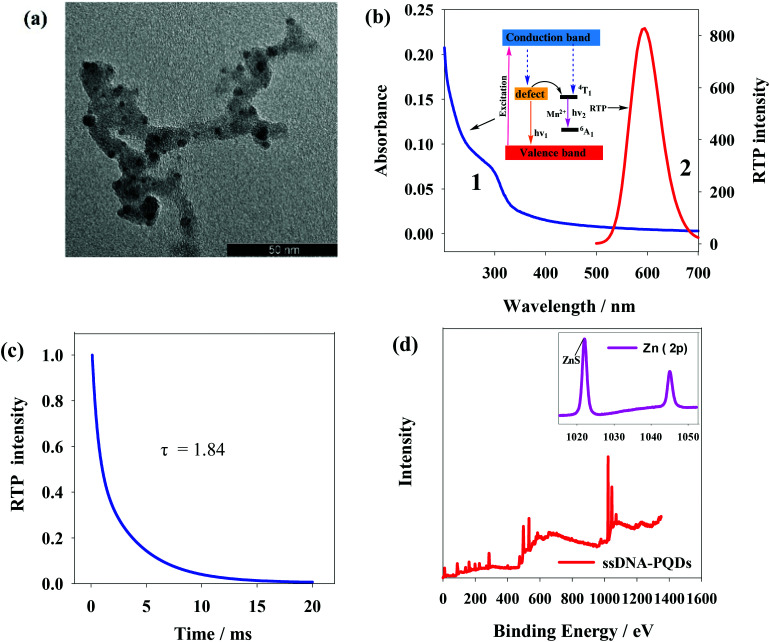
(a) TEM image of ssDNA-PQDs; (b) UV spectrum and RTP spectrum of ssDNA-PQDs; (c) the phosphorescence lifetime of ssDNA-PQDs; (d) measurement chart of XPS of ssDNA-PQDs.

Fig. 3(a) shows transmission electron microscope (TEM) graph of ssDNA-PQDs from which particle size of ssDNA-PQDs is about 3–5 nm. Fig. 3(b) shows UV spectrum and phosphorescence spectrum of ssDNA-PQDs. UV absorption peak of ssDNA-PQDs appears at 297 nm (curve 1); when excited at 297 nm, maximum phosphorescent emission peak of ssDNA-PQDs appears at 590 nm (curve 2). Phosphorescence of ssDNA-PQDs is generated through Mn^2+^ transition from triplet state (^4^T_1_) to ground state (^6^A_1_). ZnS is a kind of wide-band gap semiconductor, and its conduction band and valence band provide a wide energy range for impurity ions. When the laser is absorbed by ZnS subject, electron and cavity experience separation, and the cavity is captured by Mn^2+^, and as a result, electron and cavity are recombined on Mn^2+^, which causes Mn^2+^ excitation and releases energy in the form of phosphorescence.^32^ The absolute quantum yield of ssDNA-PQDs was 4.41%.

The phosphorescence lifetime of ssDNA-PQDs was 1.84 ms (Fig. 3(c)). X-ray photoelectron spectrometer (XPS) analysis of ssDNA-PQDs shows four intensity peaks namely 130.6 eV, 285 eV, 398.4 eV and 531.8 eV, respectively attributed to P2p, C1s, N1s and O1s (Fig. 3(d)). These results indicate that ssDNA is a constituent part of ssDNA-PQDs. 1022.3 eV and 1044.8 eV in the inset of Fig. 3(d) represent ZnS and Zn2p1 intensity peaks respectively, further indicating that ZnS crystal structure has been formed. The above results verify that ssDNA-PQDs have been successfully synthesized in this study.^33^

### 3.3 Melamine detection using ssDNA-PQDs

**Fig. 4:**
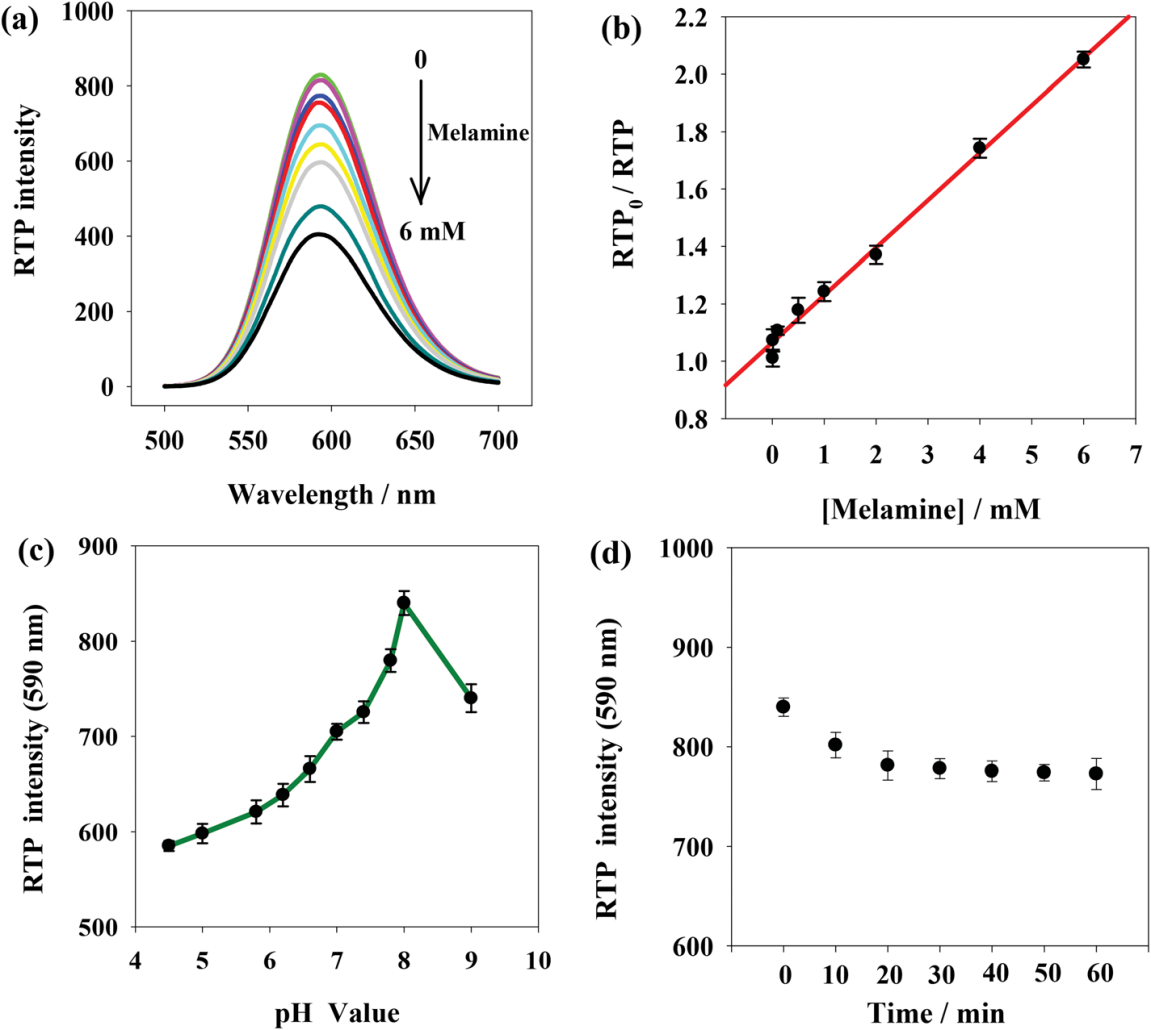
(a) The influence of melamine concentration of RTP of the ssDNA-PQDs system; (b) standard curve for melamine detection using the ssDNA-PQDs system; (c) the influence of pH value on RTP intensity in the ssDNA-PQDs and melamine reaction system; (d) the influence of reaction time on RTP intensity in the ssDNA-PQDs and melamine reaction system.

Fig. 4(a) shows RTP intensity change of ssDNA-PQDs with the addition of melamine. It can be seen that as melamine concentration gradually increased, RTP intensity of ssDNA-PQDs presented gradual regular decline (RTP quenching), so an RTP sensor which could be used for melamine detection was built in this study. Under optimal experimental conditions, RTP intensity of ssDNA-PQDs presented a favorable linear relation with melamine concentration (within the range of 0.005–6 mM) (Fig. 4(b)), and their linear equation was RTP_0_/RTP = 0.1653*c*_melamine_ + 1.0658 (*R* = 0.997), with a detection limit (3*σ*) of this method was 0.0016 mM. The solutions without melamine or with 0.005 mM melamine were each continuously detected for 11 times, and the ΔRTP relative standard deviation (RSD) was about 2.6%.

In order to improve melamine detection performance, detection conditions such as the system pH value and reaction time were optimized. According to Fig. 4(c), RTP intensity of ssDNA-PQDs was gradually enhanced when pH value gradually increased within the range of 4.5–8.0. However, when pH value increased within the range of 8.0–9.0, RTP intensity was gradually weakened, so the optimal pH value for this sensor was pH 8.0. As shown in Fig. 4(d), 20 min after melamine was added, RTP intensity was basically stable, so optimal reaction time adopted in this study was 20 min.

Though the detection limit of ssDNA-PQDs over melamine is higher compared with traditional fluorescent QDs (Table S1†), it is still lower than that of high-performance liquid chromatography (0.196 *vs.* 2 mg kg^−1^) provided in the China national standard GB/T 22388-2008. And the detection limit of melamine is far below the safety limits of melamine ingestion (20 μM in the USA and EU; 8 μM in infant formula in China).^24^ Therefore, this method can be used for the safety detection of melamine in milk. More importantly, this method based on the phosphorescence of QDs is not interfered by the background fluorescence or scattering light of biological samples and avoids complex sample preprocessing. Moreover, compared with fluorescent QDs, this method is more feasible for identification and analysis of target substances in biological samples.

### 3.4 Discussion about the interaction mechanism between ssDNA-PQDs and melamine

**Fig. 5:**
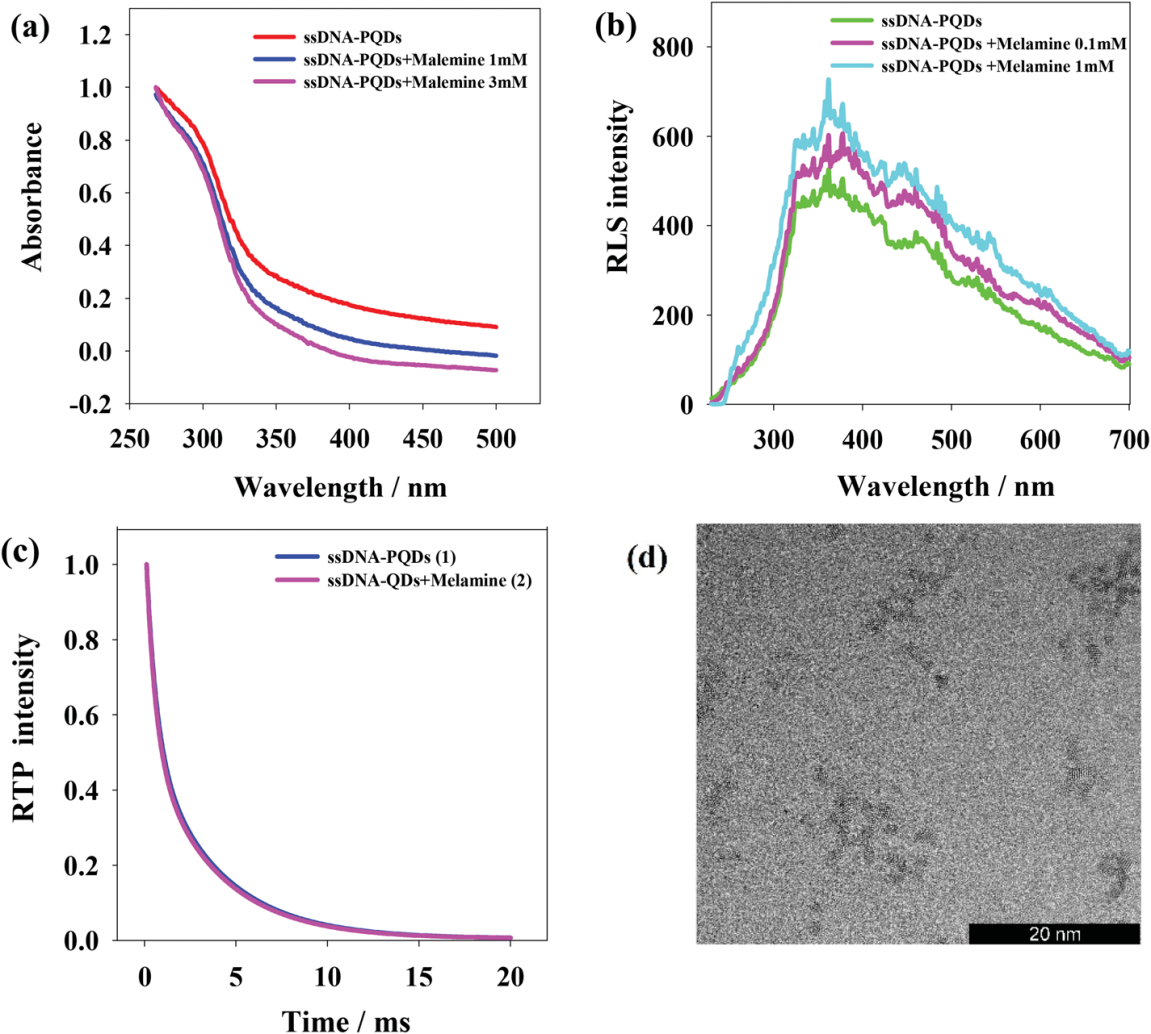
(a) UV-visible absorption spectra under the interaction between ssDNA-PQDs and melamine; (b) resonance scattering spectra under the interaction between ssDNA-PQDs and melamine; (c) the influence of melamine on ssDNA-PQDs phosphorescence lifetime; (d) TEM graph of ssDNA-PQDs added with 0.1 mM melamine.

The interaction between ssDNA-PQDs can be explained through the absorption spectrogram analysis. As shown in Fig. 5(a), three curves represent UV absorption spectra of ssDNA-PQDs, ssDNA-PQDs + melamine 1 mM and ssDNA-PQDs + melamine 3 mM respectively. It can be seen from after the addition of melamine, the UV absorption spectra of ssDNA-PQDs significantly blue shifted, and the greater the added melamine concentration, the more obvious the blue shift phenomenon, mainly because ssDNA-PQDs experienced hydrogen-bond reaction after bonding with melamine and then generated T–M–T conjugate. Meanwhile, this resulted in ssDNA-PQDs aggregation, causing PEET between ssDNA-PQDs of different particle sizes and their RTP quenching. The observed RTP quenching could be attributed to energy transfer of fluorescent particles towards a large quantity of surface defective particles, and these surface defects played trap-state roles and aggravated nonradiative energy release.^34,35^

Scattering particles formed by the two types of substances through the static effect can experience resonance light scattering (RLS). According to the RLS analysis, it can be seen that (Fig. 5(b)) single ssDNA-PQDs RLS was quite weak within the wavelength range of 200–700 nm, but ssDNA-PQDs RLS intensity would be continuously enhanced as melamine concentration increased, indicating that ssDNA-PQDs experienced aggregation during the interaction process with melamine so that larger scattering particles were formed, namely T–M–T conjugate.

Fig. 5(c) shows the phosphorescence attenuation curves before (1) and after (2) the interaction between ssDNA-PQDs and melamine. Clearly, the phosphorescence lifetime of ssDNA-PQDs was not significantly changed after the addition of melamine, indicating the quenching of ssDNA-PQDs by melamine was a static process: the melamine and ssDNA-PQDs interacted to form stable T–M–T combination. In other words, the interaction between melamine and ssDNA-PQD only quenched the intensity of QDs, but did not affect the excited or non-excited attenuation kinetics of QDs.

According to Fig. 5(d), ssDNA-PQDs went through obvious aggregation with the addition of melamine. The PEET occurring in this dense ssDNA-PQDs cluster might cause obvious UV blue shift.^34,36,37^

**Fig. 6:**
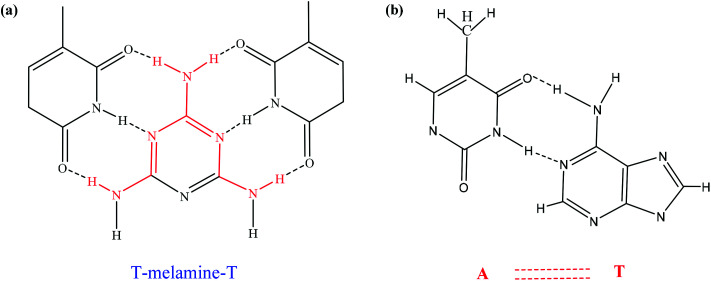
(a) Formation of T–M–T conjugate; (b) A–T pairing in DNA molecules.

The interaction between ssDNA-PQDs and melamine is shown in Fig. 6. After melamine was added into the ssDNA-PQDs solution, ssDNA-PQDs experienced hydrogen-bond interaction with melamine so as to form T–M–T conjugate (Fig. 6(a)), which caused ssDNA-PQDs aggregation, causing PEET between ssDNA-PQDs and RTP quenching. Base pairing of T–melamine–T conjugate formed in the reaction process between ssDNA-PQDs and melamine has basically identical principle with traditional basic group A–T pairing principle in DNA molecules (Fig. 6(b)).

### 3.5 Anti-interference

Interference experiments were conducted to identify how some metal ions and biomolecules commonly seen in biological fluids will affect the ssDNA-PQDs + melamine system (Table S2†). With the presence of 20 μM melamine, the RTP intensity of the system was not significantly affected by the 150-fold dosage of K^+^, 2500-fold dosage of Na^+^, 100-fold dosage of Mg^2+^, 20-fold dosage of Ca^2+^, 10-fold dosage of Zn^2+^, 0.08-fold dosage of Pb^2+^, 0.05-fold dosage of Co^2+^, 100-fold dosage of Glc, 12-fold dosage of l-Ala, 5-fold dosage of l-Lys, 2-fold dosage of l-Tyr or 10-fold dosage of l-Glu. The RTP intensity of the ssDNA-PQDs + melamine system was considerably affected by the presence of transition metals (0.001-fold dosage of Hg^2+^, 0.01-fold dosage of Ag^+^, or 0.01-fold dosage of Cu^2+^), but since Hg^2+^, Ag^+^ and Cu^2+^ are usually absent in milk or at minor concentrations, such transition metals do not affect the melamine detection in milk.

### 3.6 Analysis of practical samples

For the sake of better verification of feasibility of ssDNA-PQDs when used for melamine detection in actual biological milk samples, a recovery experiment on melamine in milk was carried out in this paper, and results (Table S3†) showed that recovery rate of melamine in milk was within 98–105.2% and relative standard deviation in the detection was smaller than 6%, indicating that this sensor system could be used for melamine detection in milk.

## 4. Conclusions

With ssDNA as the template, a novel ssDNA-PQDs nanocomposite was prepared *via* the optimized synthesis conditions. This nanocomposite was capable of direct biological functionalized modification of PQDs, and reserved the original functional sequence of ssDNA. Moreover, the specific RTP identification of melamine (T–M–T) in milk by T-bases was realized by using the principle of complementarity and the PEET. This method based on the RTP of QDs avoided the interferences from background fluorescence and scattering light in the test samples and was especially suitable for quantitative melamine detection in biological samples.

## Conflicts of interest

There are no conflicts to declare.

## Supplementary Material

RA-009-C9RA03919H-s001
