# Supplementary material for: Preparation of DNA functional phosphorescent quantum dots and application in melamine detection in milk
Source: RSC Adv. 2019 Jul 5;9(37):21147–54. doi: 10.1039/c9ra03919h (PMC9066003; doi:10.1039/c9ra03919h)
Supplement: RA-009-C9RA03919H-s001 [file RA-009-C9RA03919H-s001.pdf]

Electronic Supplementary Material (ESI) for RSC Advances.
This journal is © The Royal Society of Chemistry 2019

Supplementary Materials

Preparation of DNA functional phosphorescent quantum dots and application into melamine detection in milk

Yanming Miao*　Ruirui Wang　Xiaojie Sun　Guiqin Yan

Table of contents:

Table S1 Comparison of different methods for the determination of melamine.

Table S2 Effect of co-existing substances on the RTP intensity of 20 μM melamine.

Table S3 The results of spiked recovery test.

Shanxi Normal University, Linfen 041004, PR China. E-mail: mym8207@126.com;

Tel.: (86) 357-2051249; Fax.: (86) 357-2051243.

Table S1 Comparison of different methods for the determination of melamine.

Probe	Method	Linear range	Detection limit	Reference
DNA–Ag NCs[a]	Fluorescence	0.05-7 μM	0.01 μM	1
Ag NCs[b]	Fluorescence	0.1-30 μM	0.03 μM	2
Au NPs[c]	Fluorescence	0.05-0.5 μM	0.04 μM	3
Au NCs[d]	Fluorescence	0.5-10 μM	0.14 μM	4
CdTe QDs[e]	Fluorescence	0.79-9.5 μM	0.31 μM	5
CdS QDs	Fluorescence	2 nM-50 μM	1 μM	6
DNA (P_3)	CVG-AFS[f]	1 nM-10 μM	0. 2 nM	7
DNA (P_4)	CVG-AFS	0.1 nM-1 μM	0.02 nM	7
T_{36}DNA	Visualization	0.5-100 μM	0.08 μM	8
-	HPLC[g]	39.6-317.2 μM	0.79 μM	9
-	SERS[h]	2.5-39.6 μM	1.34 μM	10
G-quadruplex-NMM[i]	Fluorescence	0.1-100 nM	0.08 nM	11
ssDNA-PQDs	Phosphorescence	0.005-6 mM	0.0016 mM	This work

[a]DNA–Ag NCs: Oligonucleotide-stabilized silver nanoclusters; [b]Ag NCs: Ag nanoclusters; [c]Au NPs: Gold nanoparticles; [d]Au NCs: BSA-stabilized gold nanoclusters; [e]QDs: Quantum dots; [f]CVG-AFS: Chemical vapour generation coupled with atomic fluorescence spectrometry; [g]HPLC: High performance liquid chromatography; [h]SERS: Surface enhanced raman scattering; [i]G - quadruplex-NMM: N-methyl mesoporphyrin IX (NMM) and K^+ to form G-quadruplex-NMM complex.

Table S2 Effect of co-existing substances on the RTP intensity of 20 μM melamine.

Co-existing substance	[Co-existing substance] / [Melamine]	Change of the RTP Intensity (%)
K^+	150	-2.2
Na^+	2500	+1.7
Mg^{2+}	100	-3.3
Ca^{2+}	20	+3.4
Zn^{2+}	10	+3.9
Hg^{2+}	0.001	-5.6
Ag^+	0.01	-6.1
Cu^{2+}	0.01	-5.3
Pb^{2+}	0.08	+3.1
Co^{2+}	0.05	+2.1
Glc	100	-1.5
L-Ala	12	+1.9
L-Lys	5	-1.1
L-Tyr	2	+2.5
L-Glu	10	-1.1

Table S3 The results of spiked recovery test.

Sample	Added (μM)	Found (μM)	Recovery (%, n=5)	RSD (%)
Pure milk-Melamine1	10	9.8	98	4.1
Pure milk-Melamine2	50	52.6	105.2	5.7
Pure milk-Melamine3	100	98.3	98.3	3.3

1. H. Shuang, S. Zhu, Z. Liu, L. Hu, S. Parveen and G. Xu, *Biosens. Bioelectron.*, 2012, **36**, 267-270.
2. Q. Du, Q. Fei, B. Mao, S. Zhu and J. You, *New J. Chem.*, 2016, **40**, 8459-8464.
3. M. Zhang, X. Cao, H. Li, F. Guan, J. Guo, F. Shen, Y. Luo, C. Sun and L. Zhang, *Food Chem.*, 2012, **135**, 1894-1900.
4. H. Dai, Y. Shi, Y. Wang, Y. Sun, J. Hu, P. Ni and Z. Li, *Biosens. Bioelectron.*, 2014, **53**, 76-81.
5. H. Dai, S. Yan, Y. Wang, Y. Sun, J. Hu, P. Ni and L. Zhuang, *Sens. Actuators, B*, 2014, **202**, 201-208.
6. G. L. Wang, H. J. Jiao, X. Y. Zhu, Y. M. Dong and Z. J. Li, *Talanta*, 2012, **93**, 398-403.
7. P. Chen, P. Yang, R. Zhou, X. Yang, J. Chen and X. Hou, *Chem. Commun.*, 2018, **54**, 4696-4699.
8. H. Hu, J. Zhang, Y. Ding, X. Zhang, K. Xu, X. Hou and P. Wu, *Anal. Chem.*, 2017, **89**, 5101-5106.
9. G. Venkatasami and J. Sowa, *Anal. Chim. Acta*, 2010, **665**, 227-230.
10. A. Giovannozzi, F. Rolle, M. Sega, M. Abete, D. Marchis and A. Rossi, *Food Chem.*, 2014, **159**, 250-256.
11. R. Dai, X. Wang, Z. Wang, S. Mu, J. Liao, Y. Wen, J. Lv, K. Huang and X. Xiong, *Microchem. J.*, 2019, **146**, 292-299.
